# Molecular Dynamics Simulations Cyclotide Kalata B1 Interactions with Lipid Bilayers

**DOI:** 10.3390/molecules31071168

**Published:** 2026-04-01

**Authors:** Neville Y. Forlemu, Eric N. Njabon, Ajay Mallia, Simon Mwongela, Sairam Tangirala

**Affiliations:** 1 School of Science and Technology, Chemistry Program, Georgia Gwinnett College, 1000 University Center Lane, Lawrenceville, GA 30043, USA; 2 Department of Inorganic Chemistry, Faculty of Science, University of Yaoundé I, Yaoundé P.O. Box 812, Centre Region, Cameroon

**Keywords:** cyclotides, kalata B, MD simulations, peptide–lipid interactions, phospholipid bilayers, POPC, POPE, POPG

## Abstract

Cyclotides are exceptionally stable plant peptides whose biological activity is widely attributed to interactions with lipid membranes, yet the molecular mechanisms underlying these interactions remain incompletely resolved. Here, we employ microsecond-scale (1 μs) all-atom molecular dynamics simulations to investigate the membrane association of the cyclotide kalata B1 with phospholipid bilayers of distinct headgroup composition, including POPC, POPE, and POPG. This extended timescale enables full bilayer equilibration and allows observation of slower peptide-induced membrane responses that are not accessible in shorter simulations. Across all systems, kalata B1 rapidly adsorbs to the membrane surface and remains predominantly surface-associated throughout the simulations, while the cyclic cystine knot motif remains structurally intact, confirming the exceptional robustness of the cyclotide fold during membrane engagement. Lipid-dependent differences arise primarily from variations in peptide orientation, conformational flexibility, and interfacial dynamics rather than deep bilayer insertion or pore formation. Zwitterionic POPC membranes favor compact, upright peptide configurations, whereas POPE and POPG bilayers promote enhanced lateral spreading and dynamic reorganization driven by hydrogen bonding and electrostatic interactions, respectively. Leaflet-resolved analyses of lipid contacts, membrane thickness, and area per lipid reveal localized, asymmetric perturbations confined to the peptide-exposed leaflet, with no evidence of sustained bilayer thinning or global destabilization. Together, these results support an interfacial, headgroup-dependent mechanism of cyclotide membrane activity and reconcile previous experimental observations. This work provides molecular-level insight into lipid selectivity and early-stage cyclotide–membrane interactions that may inform future design of cyclotide-based bioactive agents.

## 1. Introduction

Cyclotides are small, bioactive plant peptides produced by several plant families, where they function primarily in defense against pests and pathogens [1]. These peptides are amphipathic and are distinguished by a head-to-tail cyclized backbone and six conserved cysteine residues arranged in a cyclic cystine knot (CCK) motif, which confers exceptional resistance to enzymatic degradation, chemical denaturation, and thermal unfolding [2]. This unusual stability allows cyclotides to remain biologically active under harsh environmental conditions, making them attractive candidates for pharmaceutical and agricultural applications.

**Figure 1:**
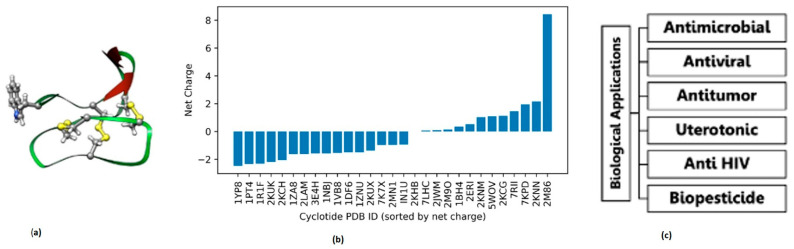
(**a**) Three-dimensional structure of cyclotide kalata B1, with color coding indicating secondary structural elements and key residues (e.g., disulfide bonds shown in yellow and structural regions highlighted accordingly). (**b**) Net charge distribution and sequence diversity of selected cyclotides based on PDB entries. (**c**) Representative biological applications of cyclotides, including antimicrobial, antiviral, antitumor, uterotonic, anti-HIV, and biopesticidal activities.

Extensive research has elucidated cyclotide structures, biosynthetic pathways, natural sources, and biological activities [3,4,5,6,7]. In particular, cyclotides display a broad spectrum of bioactivities, including antimicrobial, insecticidal, and anticancer effects (Figure 1). Their ecological role in plant defense has motivated growing interest in their development as eco-friendly alternatives to synthetic pesticides, with potential economic and environmental benefits for agriculture [6,8]. Despite this progress, the molecular mechanisms by which cyclotides interact with lipid membranes—widely believed to underlie their biological activity—remain incompletely understood [3,5].

A defining feature of cyclotides is the CCK motif, a tightly knotted network of three disulfide bonds embedded within a cyclic peptide backbone (Figure 1a) [7]. This framework imparts remarkable structural rigidity while simultaneously exposing hydrophobic and charged surface patches capable of mediating membrane interactions. Cyclotides also exhibit substantial sequence diversity outside the conserved cysteine framework (Figure 1b), suggesting that variations in surface chemistry may encode distinct membrane-binding behaviors while preserving a common structural scaffold.

Current understanding indicates that cyclotide activity is intimately linked to lipid-bilayer interactions rather than to specific protein receptors [3,9]. However, the precise nature of these interactions remains unresolved. Two canonical models are commonly invoked to describe peptide–membrane interactions. In the barrel-stave model, peptides insert perpendicularly into the bilayer, forming transmembrane pores stabilized by peptide oligomerization [10,11]. In contrast, the carpet model involves peptides binding parallel to the membrane surface, destabilizing lipid packing and inducing membrane disruption without well-defined pore formation [12]. It remains unclear whether cyclotides conform to either of these classical models or whether they employ distinct mechanisms governed by peptide sequence and lipid composition.

Experimental studies of kalata B1, the first characterized cyclotide, have provided seemingly conflicting insights. NMR and surface plasmon resonance studies suggest that kalata B1 binds primarily to the membrane surface through hydrophobic patches without deep penetration into the bilayer core [3,9,13]. Conversely, fluorescence leakage assays and cell-based studies indicate membrane permeabilization and pore-like behavior, challenging a purely surface-bound model [3]. These discrepancies highlight the limitations of experimental approaches in capturing transient, heterogeneous peptide–membrane interactions.

Adding further complexity, recent studies demonstrate that phosphatidylethanolamine (PE) headgroups play a critical role in cyclotide–membrane interactions, suggesting that lipid identity and packing properties—not simply membrane charge—are key determinants of cyclotide activity [14,15]. Moreover, it remains unclear whether cyclotides act as monomers or assemble into higher-order complexes at membrane interfaces and how such assemblies are oriented relative to the bilayer. Resolving these questions is essential for understanding why plants produce large families of cyclotides with conserved structural cores but diverse sequences.

Molecular dynamics (MD) simulations offer a powerful framework to address these unresolved questions by providing atomic-level insight into peptide–membrane interactions over extended timescales [16,17,18,19]. Unlike static structural methods, MD simulations capture peptide adsorption, reorientation, residue-specific lipid interactions, and membrane responses in real time, enabling systematic investigation of how lipid headgroup chemistry and bilayer organization modulate cyclotide behavior [19,20].

In this study, we employ microsecond-scale all-atom MD simulations to investigate the interactions of kalata B1 with phospholipid bilayers composed of 1-palmitoyl-2-oleoyl-sn-glycero-3-phosphoethanolamine (POPE), 1-palmitoyl-2-oleoyl-sn-glycero-3-phosphoglycerol (POPG), and 1-palmitoyl-2-oleoylphosphatidylcholine (POPC). These lipid systems represent zwitterionic and anionic headgroup environments relevant to mammalian and microbial membranes. The simulations aim to identify the key amino acid residues and structural motifs driving membrane association, to elucidate how lipid headgroup chemistry influences peptide orientation and interfacial interaction behavior, and to quantify the impact of cyclotide binding on membrane properties such as thickness, lipid packing, and fluidity. By integrating these analyses, this work provides a framework for interpreting kalata B1–membrane interactions and establishes a foundation for rationalizing cyclotide function in plant defense and biopesticidal applications.

## 2. Results and Discussion

**Figure 2:**
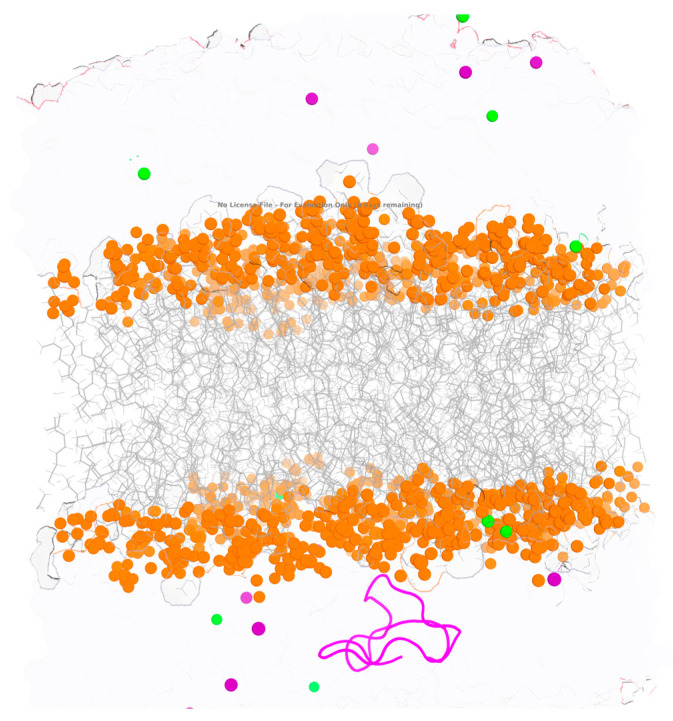
Snapshot of the simulated lipid bilayer system showing peptide–membrane interactions. Lipid phosphate groups are represented as orange spheres, lipid tails are shown as grey lines, and surrounding ions are displayed as colored spheres (green and purple). The cyclotide kalata B1 peptide is shown in magenta. The figure illustrates the peptide positioned at the membrane interface, highlighting its interfacial interaction with lipid headgroups.

This study employs a 1 μs all-atom molecular dynamics simulation to investigate the interaction of kalata B1 with mixed phospholipid bilayers composed of 1-palmitoyl-2-oleoyl-sn-glycero-3-phosphocholine (POPC), 1-palmitoyl-2-oleoyl-sn-glycero-3-phosphoethanolamine (POPE), and 1-palmitoyl-2-oleoyl-sn-glycero-3-phosphoglycerol (POPG). These phospholipids differ primarily in their headgroup chemistry and charge characteristics: POPC is zwitterionic with a choline headgroup, POPE is zwitterionic with a smaller ethanolamine headgroup, promoting tighter packing, while POPG carries a net negative charge. Such compositional heterogeneity allows assessment of how headgroup chemistry and membrane surface charge influence peptide–membrane interactions. A representative snapshot of the equilibrated bilayer system is shown in Figure 2, highlighting the spatial organization of lipid headgroups, acyl chains, and surrounding ions at the membrane–water interface. Previous computational studies of kalata B1–membrane interactions have largely relied on coarse-grained molecular dynamics simulations using DUPC/DDPC/CHOL membrane models [21], which are more representative of eukaryotic membranes but less suitable for plant-type phospholipid environments. More recent all-atom simulations have reported a preference of kalata B1 for phosphatidylethanolamine, noting selective binding and stabilization at PE-rich membranes, thereby highlighting the importance of lipid headgroup chemistry in cyclotide recognition [22]. However, these studies were conducted on shorter simulation timescales (≤200 ns) and focused primarily on interaction preference rather than membrane reorganization and long-time stability effects. By extending the simulation time to 1 μs, the present work allows sufficient equilibration of the lipid bilayers (typically requiring 300–400 ns) and captures slower lipid rearrangements and peptide-induced membrane responses that are inaccessible at shorter timescales. This longer timescale, combined with a mixed phospholipid composition, provides a more comprehensive view of how kalata B1 modulates membrane structure beyond initial binding selectivity.

### 2.1. Potential Energy and RMSD

**Figure 3:**
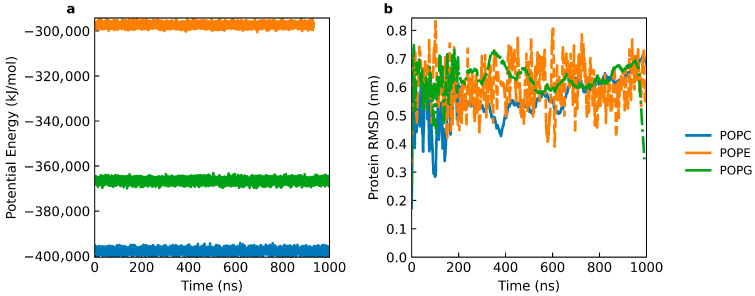
Convergence of potential Energy (**a**) and peptide RMSD (**b**), for Kalata B1 Phospholipid bilayer systems (POPC, POPG, POPE). RMSD was calculated using all peptide atoms after least-squares fitting to the initial structure.

All cyclotide–lipid bilayer systems reached equilibration within approximately 300–400 ns, as evidenced by the stabilization of both potential energy and protein root mean square deviation (RMSD) profiles (Figure 3). Following this initial relaxation from higher-energy starting configurations, the systems remained stable for the remainder of the simulations, indicating that each system attained a steady-state regime suitable for comparative analysis. The potential energy trajectories exhibit stable plateaus after equilibration, with distinct offsets observed between lipid compositions (Figure 3a). While direct comparison of absolute potential energy values across chemically distinct bilayers should be interpreted cautiously, the consistent stabilization within each system indicates well-equilibrated peptide–membrane assemblies. Analysis of peptide structural fluctuations further reveals lipid-dependent differences in cyclotide stability (Figure 3b). The POPG–cyclotide system displays the highest RMSD values (≈0.7–0.8 nm), indicating increased backbone mobility, likely reflecting enhanced electrostatic interactions with the negatively charged POPG headgroups that promote conformational rearrangements. In contrast, cyclotide RMSD values remain substantially lower in POPC and POPE bilayers (≈0.1–0.2 nm), suggesting enhanced structural stability of the peptide in zwitterionic lipid environments.

The distinct electrostatic and hydrophobic properties of phospholipid headgroups modulate cyclotide–membrane interactions. In particular, POPG-containing bilayers, bearing negatively charged headgroups, are associated with increased cyclotide conformational fluctuations, consistent with stronger electrostatic interactions that promote dynamic rearrangements rather than a single stabilized binding mode. Experimental NMR studies by Shenkarev and co-workers [9] demonstrated that the hydrophobic loops of kalata B1 play a dominant role in membrane association and interfacial stabilization, highlighting the importance of hydrophobic insertion over purely electrostatic effects. Furthermore, Henriques and co-workers showed that cyclotides exhibit enhanced membrane interaction and membrane-disruptive activity toward phosphatidylethanolamine-rich membranes, underscoring the critical role of PE headgroup chemistry in cyclotide bioactivity [14].

### 2.2. Cyclic Cystein Knot (CCK)

**Figure 4:**
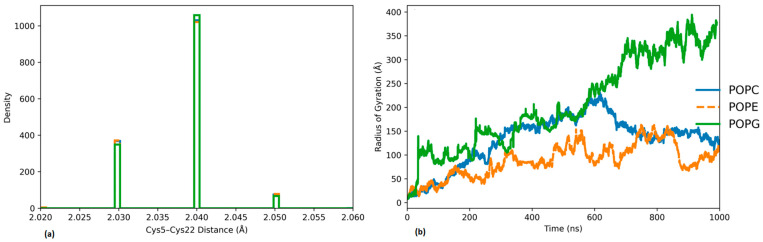
(**a**) Probability density distribution of the Cys5–Cys22 inter-residue distance within the CCK motif of kalata B1 calculated over the equilibrated simulation window (500–1000 ns) in POPC, POPE, and POPG bilayers. (**b**) Time evolution of the radius of gyration (Rg) of kalata B1 during the 1 μs simulation in the different lipid environments.

To quantify the stability of the Cys5–Cys22 disulfide bridge within the CCK motif, the probability distribution of the inter-residue distance was calculated over the equilibrated portion of the trajectory (500–1000 ns) (Figure 4). All membrane systems display a narrow unimodal distribution centered at approximately 2.03–2.05 nm, indicating preservation of the disulfide-constrained fold during membrane interaction. The limited spread of the distribution suggests minimal structural perturbation of the CCK core, irrespective of lipid composition.

The radius of gyration (Rg) was used to evaluate lipid-dependent changes in cyclotide compactness and conformational dynamics (Figure 4b). Distinct behaviors were observed across the three membrane environments. In the presence of POPE, kalata B1 maintained a relatively constant Rg throughout the simulation, indicating a compact and stable conformation. Simulations with POPC showed a modest increase in Rg over time, suggesting limited conformational rearrangements involving non-cysteine residues at the membrane interface. In contrast, the POPG system exhibited the largest increase and fluctuations in Rg, reflecting enhanced conformational expansion and dynamic rearrangement of the peptide while preserving the structurally rigid disulfide-bonded core.

**Figure 5:**
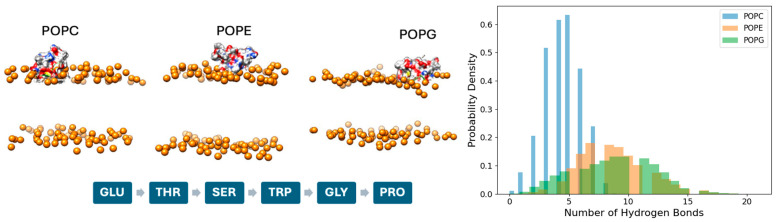
Representative snapshots of kalata B1 at the membrane interface in POPC, POPE, and POPG bilayers (**Left**). Lipid headgroups are shown as orange spheres, while the peptide is displayed in atomic representation (oxygen in red, nitrogen in blue, and carbon in white/grey). The upper and lower panels correspond to the two leaflets of the bilayer. The sequence of key interacting residues is indicated below. (**Right**) Probability distribution of the number of hydrogen bonds formed between the peptide and lipid headgroups, shown for POPC (blue), POPE (orange), and POPG (green).

Throughout the simulations, the cyclic cystine knot (CCK) motif of kalata B1 remained structurally intact, as evidenced by the preservation of inter-cysteine distances, consistent with the well-established experimental stability of cyclotides [23,24]. These results confirm that membrane association does not compromise the disulfide-bonded core, allowing the peptide to retain its characteristic bioactive fold across different lipid environments. While the CCK motif provides a rigid structural scaffold, lipid-dependent differences were observed in peptide positioning and interfacial engagement (Figure 5). In POPC bilayers, kalata B1 remains predominantly surface-associated with limited penetration into the headgroup region, consistent with weaker electrostatic interactions. In contrast, POPE and especially POPG systems display closer peptide–headgroup proximity and more extensive interfacial contact, reflecting enhanced hydrogen bonding and electrostatic contributions. These trends are supported by the time-resolved hydrogen bond analysis (Figure 5, right panel), where POPG exhibits the highest number of peptides–lipid hydrogen bonds, followed by POPE, while POPC shows comparatively fewer interactions. The increased hydrogen bonding in POPG correlates with stronger electrostatic attraction between the anionic headgroups and charged residues of the peptide, promoting greater surface stabilization and conformational adaptability.

Inspection of residue exposure further indicates lipid-dependent modulation of key side chains, including Glu, Thr, Ser, Trp, Gly, and Pro, at the membrane interface. Notably, charged and polar residues contribute to headgroup interactions, while hydrophobic residues facilitate interfacial anchoring without deep insertion into the bilayer core. This behavior is consistent with experimental and biophysical studies of membrane-active peptides, where electrostatic and hydrophobic forces jointly govern peptide orientation, dynamics, and functional activity [25].

### 2.3. Dynamic Interaction of Kalata B1 with Lipid Bilayers

**Figure 6:**
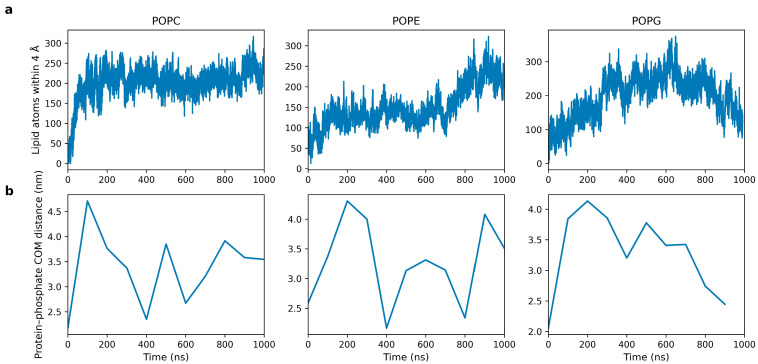
(**a**). Cyclotide and lipid bilayer contacts (POPC, POPE, and POPG). (**b**) the distance between Kalata B1 and the phosphate groups of a lipid bilayer.

The dynamic association of kalata B1 with lipid bilayers was quantified by monitoring lipid contact frequencies and peptide–membrane distances throughout the simulation (Figure 6). The number of lipid atoms within 4 Å of the peptide provides a measure of interfacial engagement and surface residency over time. POPC bilayers exhibit the highest average number of surrounding lipid atoms (approximately 250–280), indicating persistent surface association at the densely packed zwitterionic headgroup region. In contrast, POPG and POPE systems display lower average contact numbers (approximately 150–200 and 120–170, respectively), accompanied by greater temporal variability. These differences reflect variations in headgroup packing density, electrostatic environment, and peptide mobility at the membrane interface rather than differences in overall peptide-membrane association behavior.

Consistent with these trends, the distance between the peptide center of mass and lipid phosphate groups (Figure 6b) remains within close proximity to the membrane surface across all systems, fluctuating between approximately 2.0 and 4.8 nm throughout the trajectory. In POPC bilayers, the COM–phosphate distance stabilizes at shorter values at later times, consistent with sustained surface adsorption. By contrast, POPE and POPG systems exhibit larger distance fluctuations, indicative of dynamic reorientation and lipid-dependent interfacial rearrangements rather than repeated detachment events.

Together, these metrics indicate that kalata B1 interacts predominantly through surface adsorption and interfacial anchoring, with lipid headgroup chemistry modulating peptide mobility and membrane engagement. No evidence of deep insertion or stable pore formation was observed within the simulation timescale, suggesting an interfacial interaction mode due to electrostatic contacts and hydrophobic loop anchoring. These findings are partially consistent with experimental observations indicating that cyclotide activity depends strongly on lipid headgroup composition, particularly the presence of phosphatidylethanolamine (PE), which has been shown to enhance membrane binding and perturbation effects [14,26]. The present simulations can also be interpreted in the context of previous experimental studies investigating cyclotide membrane activity. Experimental work by Henriques et al. [26] demonstrated that kalata B1 can enter cells through interactions with phosphatidylethanolamine-containing membranes, suggesting that PE lipids play a critical role in mediating cyclotide–membrane interactions. Similarly, Cranfield et al. [27] reported that kalata B1 exhibits surfactant-like activity in PE-rich lipid membranes and can induce membrane destabilization in model vesicle systems. The predominantly surface-associated interactions observed in the present simulations may appear to differ from these experimental observations. However, the simulations performed here examine the interaction of a single cyclotide molecule (kalata B1) with a model lipid bilayer, whereas experimental studies typically involve multiple peptides interacting cooperatively with membranes. Membrane disruption mechanisms reported experimentally are therefore likely to arise from collective peptide behavior, including aggregation or cooperative insertion events that cannot be captured in single-peptide simulations. In addition, the simulations probe dynamics on microsecond timescale, whereas membrane permeabilization and peptide-induced membrane remodeling observed experimentally may occur over longer timescales. The interactions observed here therefore likely represent the early stages of cyclotide–membrane association, including adsorption, orientation, and lipid headgroup engagement. These early events may precede the cooperative membrane perturbation mechanisms observed experimentally. Importantly, the simulations nevertheless reproduce lipid-dependent differences in peptide–membrane interactions. In particular, phosphatidylethanolamine-containing membranes display increased peptide–lipid hydrogen bonding and greater interfacial lipid rearrangements relative to POPC systems. These observations are consistent with experimental studies highlighting the role of PE lipids in cyclotide membrane recognition. These findings highlight an apparent discrepancy with experimental studies reporting membrane permeabilization and pore-like behavior. However, this difference can be rationalized by considering the distinct conditions probed. The present simulations involve a single peptide and capture early-stage membrane association on the microsecond timescale, whereas experimental observations likely reflect cooperative interactions of multiple peptides and longer-timescale membrane remodeling processes.

Therefore, rather than contradicting experimental results, our simulations provide a molecular-level description of early-stage adsorption and interfacial binding events that precede collective membrane disruption. Importantly, while we observe agreement with previous studies regarding lipid-dependent binding, particularly the role of phosphatidylethanolamine, we also explicitly acknowledge that our results do not reproduce the full membrane-disruptive behavior reported experimentally. This distinction clarifies the complementary nature of MD simulations and experimental observations.

A limitation of the present study is that only a single peptide was simulated in each system; future work including multiple peptides or enhanced sampling approaches may provide additional insight into cooperative membrane disruption mechanisms.

While independent replicate simulations would provide additional statistical robustness, microsecond-scale trajectories have been shown in previous MD studies to capture stable peptide–membrane interaction modes and equilibrated interfacial behavior. In the present study, convergence of key observables—including RMSD, peptide–membrane distance, and lipid contact profiles—supports that the reported interaction patterns are representative of stable interaction regimes within the simulated timescale.

### 2.4. Membrane Thickness and Area per Lipid

**Figure 7:**
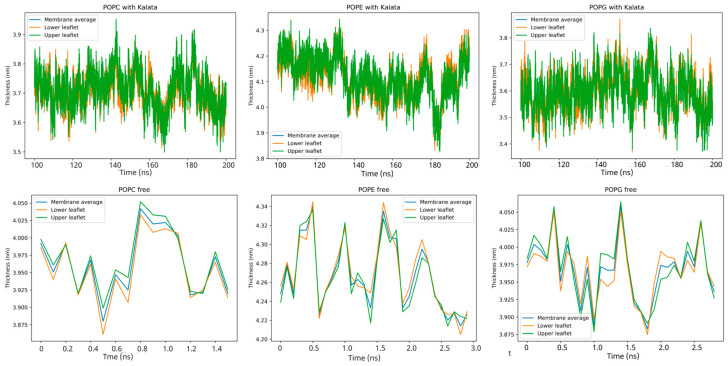
Time evolution of bilayer thickness for POPC, POPE, and POPG membranes in the presence (**top panels**) and absence (**bottom panels**) of kalata B1. Thickness values are shown for the membrane average (blue) and individual lower and upper leaflets (orange and green). Peptide–bilayer systems were simulated for 200 ns, while shorter simulations were performed for peptide-free bilayers as reference controls. Thickness fluctuations represent equilibrated membrane dynamics over the analyzed trajectories.

Membrane thickness and area per lipid are key parameters governing bilayer structural integrity and responsiveness to peptide association. Analysis of membrane thickness in POPC, POPE, and POPG bilayers, both in the absence and presence of kalata B1 (Figure 7), reveals modest but clearly lipid-dependent structural responses to peptide binding. In pure bilayers, all three lipid systems exhibit comparable thickness values within expected ranges. Upon interaction with kalata B1, small yet measurable fluctuations in membrane thickness are observed across all systems, with POPE displaying the largest variability (approximately 3.4–3.8 nm), while POPC and POPG show more moderate changes. Importantly, these thickness variations remain largely comparable between the upper and lower leaflets and do not indicate sustained bilayer thinning or global destabilization. Instead, the observed fluctuations point to elastic, composition-dependent membrane adjustments arising from surface-associated peptide interactions. The heightened sensitivity of PE-rich membranes is consistent with their intrinsic lipid packing properties and enhanced hydrogen-bonding capacity, which make them more responsive to interfacial perturbations. Together, these observations support a model in which kalata B1 influences lipid packing and local membrane properties through surface adsorption and interfacial anchoring rather than pore formation, providing a mechanistic interpretation for lipid-dependent interaction patterns and membrane recognition relevant to cyclotide biological activity.

**Figure 8:**
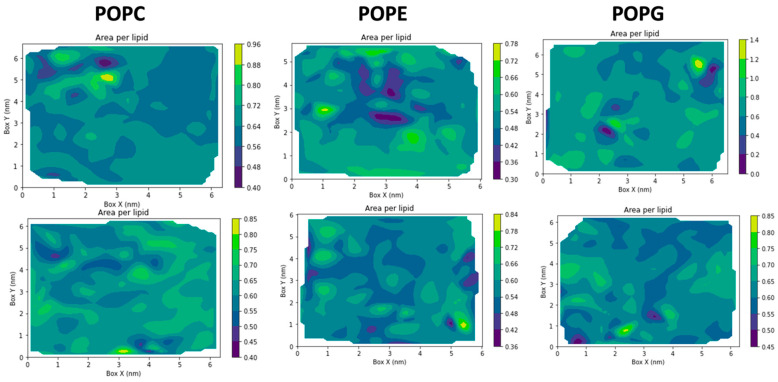
Heatmap representation of the average area per lipid in lower leaflets of lipid bilayers (**bottom row**) and bilayers in the presence of Kalata B1 (**top row**) for different phospholipid compositions (POPC, POPE, and POPG). The time axis corresponds to the sampling window used for generating the heatmap representation and does not reflect the full simulation duration.

The color-coded heatmaps in Figure 8 depict the spatial distribution of area per lipid headgroup across the upper (top row) and lower (bottom row) leaflets of POPC, POPE, and POPG bilayers. Variations in color intensity reflect local differences in lipid packing, with warmer colors (yellow–green) indicating regions of increased area per lipid (lipid expansion) and cooler colors (blue–purple) corresponding to more compact lipid arrangements. Across all bilayer compositions, both leaflets exhibit heterogeneous yet continuous packing landscapes, characterized by localized regions of lipid expansion and compression rather than uniform, large-scale reorganization. Clear lipid- and leaflet-dependent differences emerge upon peptide binding. In POPC bilayers, the spatial patterns in the upper and lower leaflets are highly similar, indicating a largely symmetric membrane response consistent with elastic surface adsorption of kalata B1. In contrast, POPE and POPG membranes display pronounced leaflet asymmetry, with the peptide-exposed upper leaflet exhibiting enhanced spatial heterogeneity and more frequent localized expansion relative to the lower leaflet. This asymmetry is most pronounced in POPG, where electrostatic interactions between the anionic headgroups and the cyclotide promote localized lipid rearrangements at the interface. The persistence of comparatively uniform patterns in the lower leaflet across all systems indicates that peptide-induced perturbations remain confined to the interfacial region and do not propagate across the bilayer thickness.

Quantitative image-based analysis further supports these observations. The fraction of high-intensity regions (green–yellow) in the upper leaflet increases progressively from POPC (6.3%) to POPE (8.5%) and is highest in POPG membranes (10.4%), while the lower leaflet consistently exhibits weaker and less variable responses across all lipid types. This gradient highlights a lipid headgroup–dependent modulation of membrane packing and reinforces a preferential surface association of kalata B1 with PE- and PG-containing membranes.

Together, these spatial and quantitative analyses are consistent with a leaflet-specific, surface-associated interaction mechanism, in which kalata B1 induces localized lipid packing rearrangements at the peptide-exposed leaflet without causing global bilayer destabilization. Such behavior is consistent with an interfacial activity model driven by lipid headgroup chemistry, rather than deep insertion or pore formation, and provides a structural basis for the lipid selectivity and membrane activity observed for cyclotides

## 3. Computational Method

In this study, unconstrained all-atom 1-μs molecular dynamics (MD) simulations were performed using the GROMACS software package (version 2019.6) to investigate the interactions between the cyclotide kalata B1 (PDB ID: 1KAL) and three lipid bilayers composed of distinct phospholipid species [27]. The initial (NMR) structure of Kalata B1 was downloaded from the protein data bank with accession code 1kal [28,29]. Three phospholipids, representing neutral and negatively charged head groups, were selected to construct the bilayers to mimic microbial lipid bilayers: These include: 1-palmitoyl-2-oleoyl-sn-glycero-3-phosphoethanolamine (POPE), 1-palmitoyl-2-oleoyl-sn-glycero-3-phosphoglycerol (POPG), and 1-palmitoyl-2-oleoylphosphatidylcholine (POPC).

The CHARMM-GUI interface and GROMACS were used to build, solvate, and equilibrate the molecular systems [30,31]. The simulations employed the CHARMM-modified TIP3P water model (CHARMM TIP3P), consistent with the CHARMM36 force field distributed via CHARMM-GUI. The CHARMM36a force field, specifically parameterized for lipid bilayers and protein-lipid interactions, was applied to describe intermolecular and intramolecular forces [32,33,34]. Each cyclotide-lipid system was confined to an orthorhombic simulation box and solvated with 15–20 molecular layers of water using the TIP3P water model [35,36]. Sodium and chloride counterions were added to neutralize the system. The Verlet algorithm was employed to integrate Newton’s equations of motion, and the NPT ensemble was used to maintain a constant temperature (310 K) and pressure. MD simulations were performed with long microsecond-scale trajectories using a 2-fs time step to accurately capture cyclotide-lipid interactions over extended timescales. Details of the cyclotide-lipid bilayer system is summarized in Table 1 below.

Long-timescale molecular dynamics simulations were performed to (i) characterize the spatial distribution and membrane association behavior of the cyclotide in solution, (ii) identify local molecular interactions driving peptide–bilayer complex formation, and (iii) evaluate lipid bilayer conformational changes during the simulation. In addition, structural variations within the cyclic cystine knot (CCK) motif were analyzed by calculating the root mean square deviation (RMSD) and root mean square fluctuation (RMSF). RMSD was computed using all peptide atoms after least-squares fitting to the initial structure to remove overall translational and rotational motion, enabling differentiation between rigid and flexible peptide regions during membrane interaction.

Artificial intelligence tools (ChatGPT, based on the GPT-5.3 model OpenAI) were used to assist in the interpretation and refinement of the textual description of selected figure data. The AI tools did not generate data, perform calculations, or influence the scientific conclusions. All analyses and interpretations were conducted and validated by the authors. No independent replicate simulations were performed; therefore, the results are interpreted qualitatively based on consistent trends observed over the equilibrated trajectory rather than statistical averaging across multiple runs.

## 4. Conclusions

This study employed microsecond-scale all-atom molecular dynamics simulations to investigate the interaction of the cyclotide kalata B1 with model phospholipid bilayers composed of POPC, POPE, and POPG. Across all systems, the peptide remains predominantly associated with the membrane interface rather than inserting deeply into the bilayer core. Analyses of peptide–lipid contacts, hydrogen bonding, peptide–membrane distances, and lipid packing perturbations consistently indicate that kalata B1 engages membranes through surface adsorption and interfacial anchoring.

The simulations reveal clear lipid-dependent differences in interfacial interaction behavior. In particular, POPE- and POPG-containing bilayers exhibit increased peptide–lipid contacts, hydrogen bonding, and dynamic interfacial rearrangements relative to POPC membranes, highlighting the role of lipid headgroup chemistry in modulating peptide orientation and membrane engagement. These observations are partially consistent with experimental studies that emphasize the importance of phosphatidylethanolamine in cyclotide activity.

At the same time, the present simulations do not reproduce membrane permeabilization or pore-like behavior reported experimentally. This apparent discrepancy can be rationalized by differences in system conditions: the simulations involve a single peptide and capture early-stage interactions on the microsecond timescale, whereas experimental observations likely reflect cooperative effects involving multiple peptides and longer-timescale membrane remodeling processes. The results obtained here therefore represent early events in cyclotide–membrane association, including adsorption, orientation, and lipid headgroup engagement, which may precede collective membrane disruption.

While the microsecond simulation timescale allows for equilibration of peptide–membrane interactions and convergence of key observables, no independent replicate simulations were performed. Consequently, the findings should be interpreted as qualitative, atomistic insights into interfacial interaction behavior rather than definitive mechanistic conclusions. Future work incorporating multiple peptides, replicate simulations, and enhanced sampling approaches will be necessary to fully resolve cooperative membrane disruption mechanisms and quantify interaction energetics.

Overall, this study provides a detailed molecular-level description of early-stage cyclotide–membrane interactions and offers a consistent framework for interpreting how lipid composition influences peptide behavior at membrane interfaces.

## Figures and Tables

**Table 1:** Details of cyclotide lipid bilayer systems simulated.

Molecules	128 POPC	128 POPE	128 POPG
Kalata B1(residues/atoms)	29/388	29/388	29/388
Water molecules	7282	5783	7019
Na ions	10	10	128
Cl ions	0	10	10
Total atoms	21,846	17,349	21,057

## Data Availability

The data supporting the findings of this study, including molecular dynamics simulation trajectories and analysis files, are available from the corresponding author upon reasonable request.

## Abbreviations

The following abbreviations are used in this manuscript:

CCK	Cyclic cystine knot
MD	Molecular dynamics
PE	Phosphatidylethanolamine
POPC	1-palmitoyl-2-oleoylphosphatidylcholine
POPE	1-palmitoyl-2-oleoyl-sn-glycero-3-phosphoethanolamine
POPG	1-palmitoyl-2-oleoyl-sn-glycero-3-phosphoglycerol
RMSD	Root mean square deviation
